# Pericardial Fat and Myocardial Perfusion in Asymptomatic Adults from the Multi-Ethnic Study of Atherosclerosis

**DOI:** 10.1371/journal.pone.0028410

**Published:** 2011-12-09

**Authors:** Tina E. Brinkley, Michael Jerosch-Herold, Aaron R. Folsom, J. Jeffrey Carr, W. Gregory Hundley, Matthew A. Allison, David A. Bluemke, Gregory L. Burke, Moyses Szklo, Jingzhong Ding

**Affiliations:** 1 Department of Internal Medicine, Wake Forest School of Medicine; Winston-Salem, North Carolina, United States of America; 2 Department of Radiology, School of Medicine, University of Minnesota, Minneapolis, Minnesota, United States of America; 3 Division of Epidemiology and Community Health, School of Public Health, University of Minnesota, Minneapolis, Minnesota, United States of America; 4 Department of Radiology, Wake Forest School of Medicine, Winston-Salem, North Carolina, United States of America; 5 Department of Family and Preventive Medicine, University of California San Diego, La Jolla, California, United States of America; 6 National Institutes of Health Clinical Center and National Institute of Biomedical Imaging and Bioengineering, Bethesda, Maryland, United States of America; 7 Division of Public Health Sciences, Wake Forest School of Medicine, Winston-Salem, North Carolina, United States of America; 8 Department of Epidemiology, Johns Hopkins University, Baltimore, Maryland, United States of America; Innsbruck Medical University, Austria

## Abstract

**Background:**

Pericardial fat has adverse effects on the surrounding vasculature. Previous studies suggest that pericardial fat may contribute to myocardial ischemia in symptomatic individuals. However, it is unknown if pericardial fat has similar effects in asymptomatic individuals.

**Methods:**

We determined the association between pericardial fat and myocardial blood flow (MBF) in 214 adults with no prior history of cardiovascular disease from the Minnesota field center of the Multi-Ethnic Study of Atherosclerosis (43% female, 56% Caucasian, 44% Hispanic). Pericardial fat volume was measured by computed tomography. MBF was measured by MRI at rest and during adenosine-induced hyperemia. Myocardial perfusion reserve (PR) was calculated as the ratio of hyperemic to resting MBF.

**Results:**

Gender-stratified analyses revealed significant differences between men and women including less pericardial fat (71.9±31.3 vs. 105.2±57.5 cm^3^, p<0.0001) and higher resting MBF (1.12±0.23 vs. 0.93±0.19 ml/min/g, p<0.0001), hyperemic MBF (3.49±0.76 vs. 2.65±0.72 ml/min/g, p<0.0001), and PR (3.19±0.78 vs. 2.93±0.89, p = 0.03) in women. Correlations between pericardial fat and clinical and hemodynamic variables were stronger in women. In women only (p = 0.01 for gender interaction) higher pericardial fat was associated with higher resting MBF (p = 0.008). However, this association was attenuated after accounting for body mass index or rate-pressure product. There were no significant associations between pericardial fat and hyperemic MBF or PR after multivariate adjustment in either gender. In logistic regression analyses there was also no association between impaired coronary vasoreactivity, defined as having a PR <2.5, and pericardial fat in men (OR, 1.18; 95% CI, 0.82–1.70) or women (OR, 1.11; 95% CI, 0.68–1.82).

**Conclusions:**

Our data fail to support an independent association between pericardial fat and myocardial perfusion in adults without symptomatic cardiovascular disease. Nevertheless, these findings highlight potentially important differences between asymptomatic and symptomatic individuals with respect to the underlying subclinical disease burden.

## Introduction

Visceral adiposity is a well-established risk factor for cardiovascular morbidity and mortality [1]. Pericardial fat is a visceral fat depot adjacent to the myocardium and coronary arteries that may be particularly relevant for cardiovascular diseases [2]. We and others have shown that pericardial fat is increased in coronary artery disease (CAD) patients and is positively associated with disease severity [3], [4]. We also reported that higher pericardial fat is associated with higher amounts of coronary calcium, even after adjusting for total and abdominal obesity [5]. Given its close proximity to the coronary arteries (<100 µm), as well as the pericardial, pericardiophrenic, and musculophrenic arteries, it is thought that pericardial fat interacts with neighboring cells through the release of bioactive factors [6]. Indeed, pericardial fat around the coronary arteries has been shown to express relatively high levels of interleukin-6, tumor necrosis factor alpha, and monocyte chemoattractant protein-1, but low levels of adiponectin [7], [8]. This heightened pro-inflammatory state may promote endothelial dysfunction and vascular remodeling [9], [10]. Moreover, increased adiponectin levels in the coronary circulation have been associated with a greater coronary vasodilatory response [11]. Taken together, these data suggest that pericardial fat may have both direct and indirect effects on vascular structure and function.

Very few studies have explored the *in vivo* relationship between coronary vasodilatory function and pericardial fat. In the clinical setting, the measurement of blood flow through the coronary arteries (i.e. myocardial perfusion) by non-invasive imaging is often used to assess coronary vasoreactivity [12]. Recently, Tamarappoo et al. reported that among individuals without known CAD, pericardial fat volume was 22% higher in those with myocardial ischemia compared to non-ischemic controls [13]. The study population included both symptomatic and asymptomatic individuals. Other studies in adults with chest pain have found similar relationships between cardiac obesity and myocardial perfusion [14], [15]. However, based on the literature to date, it is unclear if pericardial fat adversely affects the coronary microcirculation in asymptomatic individuals. Thus, the purpose of this study was to investigate the association between pericardial fat and myocardial perfusion in asymptomatic adults free of cardiovascular disease from the Multi-Ethnic Study of Atherosclerosis (MESA). As coronary vasoreactivity may be impaired early in the atherosclerotic process, even in the absence of ischemic symptoms [12], asymptomatic persons likely have a different subclinical disease burden compared to symptomatic persons. We previously reported that a number of CAD risk factors, including older age, male gender, elevated blood pressure, and high cholesterol levels, are associated with coronary vascular dysfunction in the MESA cohort, as evidenced by lower myocardial blood flow (MBF) and/or myocardial perfusion reserve (PR) [16], [17]. In the present analysis, we hypothesized that impaired coronary vasoreactivity would also be associated with a higher pericardial fat volume in this asymptomatic population.

## Materials and Methods

### Ethics Statement

Institutional Review Board approval was obtained at all MESA sites (Northwestern University, Wake Forest University, Johns Hopkins University, Columbia University, University of Minnesota, and UCLA). All study procedures were in accordance with institutional guidelines, and all participants provided written informed consent.

#### Study Population

MESA is a prospective community-based cohort study of 6,814 men and women aged 45 to 84 years from four different ethnic groups (Caucasian, African American, Hispanic, and Chinese) [18]. Participants were recruited between July 2000 and September 2002 from six field centers including Forsyth County, NC; Northern Manhattan and the Bronx, NY; Baltimore City and Baltimore County, MD; St. Paul, MN; Chicago, IL; and Los Angeles County, CA. Individuals were excluded if they had physician-diagnosed heart attack, angina, stroke, transient ischemic attack, heart failure, or atrial fibrillation; were taking nitroglycerin; or had undergone coronary artery bypass grafting, angioplasty, valve replacement, pacemaker or defibrillator implantation, or any surgery on the heart or arteries. Each participant at the Minnesota field center (n = 1,066) was contacted for a perfusion study either immediately after the baseline MESA exam or later by mail. Of those, 234 agreed to participate. The present analysis is based on data from 214 participants after excluding those with missing data for MBF (n = 5) or pericardial fat (n = 8) and those who took caffeine within 12 hours of the MRI examination (n = 7). Except for a lower prevalence of hypertension (29.9% vs. 38.5%), this subset had similar characteristics to the individuals who declined to participate in the perfusion study or were excluded from the analyses.

#### Pericardial Fat

Pericardial fat volume was measured from computed tomography (CT) scans performed at the baseline MESA exam [5]. Our measurement of pericardial fat includes both epicardial fat (located within the pericardium) and paracardial fat (located superficial to the pericardium). We and others have shown an excellent correlation between pericardial and epicardial fat depots (r = 0.92 and 0.97, respectively) [4], [19]. Given the lower reproducibility of epicardial fat measurements [19], [20] and the difficulty in visualizing the pericardium, particularly over the left ventricle [21], we chose to measure only pericardial fat for these analyses. Segmentation was achieved by isolating pericardial fat and heart from the thorax using specific anatomic landmarks. The anterior border of the volume was defined by the chest wall and the posterior border by the aorta and the bronchus. Slices within 15 mm above and 30 mm below the superior extent of the left main coronary artery (a total of 19 slices) were included in the analysis. This region of the heart was selected because it includes the pericardial fat located specifically around the proximal coronary arteries (left main coronary, left anterior descending, right coronary, and circumflex arteries). Volume Analysis software (GE Healthcare, Waukesha, WI) was used to discern fat from the remaining portions of the heart with a threshold of −190 to −30 Hounsfield units. The volume was the sum of all voxels containing pericardial fat. Intra-class correlation coefficients for inter-reader and intra-reader reliability are 0.997 and 0.999, respectively [5]. This measurement is highly correlated with the “gold standard” method (r = 0.93) which measures the total volume of pericardial fat encasing the heart and takes about half the time to complete [5]. Pericardial fat was assessed as the total absolute volume and indexed to left ventricular mass (i.e. pericardial fat index).

#### MRI Perfusion Study

Cardiac MRI was performed with a 1.5-T clinical MR scanner (Sonata, Siemens Medical Systems, Iselin, New Jersey) an average of 334 days (range: 20–645) after the baseline examination. Participants were asked to refrain from caffeine for 12 hours before this visit. During the exam, participants were positioned supine with a flexible, four-element phased-array coil placed over their heart, with two elements of a spine array coil serving as posterior antennae. Starting at the third or fourth heartbeat, 0.04 mmol/kg body weight of a Gd-DTPA contrast agent (Magnevist, Berlex, Wayne, NJ) was administered intravenously at a rate of 7 ml/s. T1-weighted gradient-echo imaging of 2 to 3 adjacent left ventricle slices in the short axis orientation, with a nonslice-selective saturation recovery magnetization preparation, was used to visualize the first pass of the injected contrast bolus through the heart, as previously described [22]. To induce vasodilation, 0.14 mg/kg/min of adenosine was infused intravenously for 3 minutes before the start of the scan, blocked for approximately 3 seconds during MR contrast injection, resumed immediately thereafter, and then discontinued 10 to 15 seconds after starting the perfusion scan. A first perfusion scan was performed at rest, followed by a second scan approximately 15 minutes later during maximal vasodilation. Blood pressure, heart rate, and an electrocardiogram were monitored and recorded during the exam. Rate-pressure product (RPP) was calculated as the product of heart rate and systolic blood pressure divided by 10,000.

#### Image Analysis and MBF Quantification

Endocardial and epicardial contours were manually traced. The myocardium was subdivided into eight transmural sectors of equal circumferential extent along the myocardial centerline. Region-of-interest signal intensity curves were generated with the MASS CMR image analysis software (Laboratory for Clinical and Experimental Image Processing, Leiden University, The Netherlands). These curves represent the change of mean signal intensity as a function of time, corrected for baseline- and coil-sensitivity variations. In accordance with the central volume principle [23], MBF (in ml/min/g) was estimated from the initial amplitude of the myocardial impulse response by deconvolution analysis of the myocardial signal intensity curves. Custom-written software was used to perform a model-independent deconvolution of the signal intensity curves, with an arterial input measured in the center of the left ventricle. As described and validated previously, MBF estimation by this method is highly correlated with measurements using radioisotope-labeled microspheres (R^2^ = 0.995), which is the gold-standard in MBF quantification [24]–[26]. All MBF measurements are reported as global averages over the eight myocardial segments and two to three slices. The intra-class correlation for duplicate global MBF measurements taken ∼1 year apart are 0.65 for resting MBF and 0.71 for hyperemic MBF [22]. Myocardial PR was calculated as the ratio of hyperemic to resting MBF. An index of coronary vascular resistance (CVR) was calculated as mean arterial pressure divided by MBF.

#### Clinical Variables from the Baseline MESA Exam

Standard questionnaires were used to collect information on demographics, smoking, comorbidities, and medications. Height and weight were measured and body mass index (BMI) was calculated as height divided by weight squared. Waist circumference (at the umbilicus) and hip circumference (at the maximum circumference of the buttocks) were measured using a Gulick II measuring tape. Seated blood pressure was measured in the right arm after five minutes of rest using a Dinamap model Pro 100 automated oscillometric sphygmomanometer (Critikon, Tampa, FL). Hypertension was defined as systolic blood pressure ≥140 mmHg, diastolic blood pressure ≥90 mmHg, self-reported history of hypertension, or current use of anti-hypertensive medications. Diabetes was defined as fasting glucose ≥126 mg/dl, self-reported history of diabetes, or current use of diabetes medications. Fasting blood samples were analyzed at a central laboratory using standard methods to determine low-density lipoprotein (LDL) and high-density-lipoprotein (HDL) cholesterol. C-reactive protein was measured using the BNII nephelometer (Dade Behring Inc, Deerfield, IL). Coronary calcium was measured by electron-beam or four-detector row CT, as previously described [27]. The amount of calcium averaged from two consecutive scans was quantified using the Agatston scoring method [28]. Left ventricular mass was determined by volumetric imaging [29].

#### Statistical Analysis

All statistical analyses were performed using SAS software version 9.1 (SAS Institute, Cary, NC). Chi-square tests and analysis of variance were used to determine differences in categorical and continuous variables, respectively. Spearman correlation coefficients were used to describe the relationship of pericardial fat with clinical and hemodynamic variables. Linear regression was used to determine the relationship of pericardial fat with resting MBF, hyperemic MBF, and PR. We also modeled hyperemic MBF with adjustment for resting MBF as an alternative interpretation of PR. Covariates were included based on univariate associations with pericardial fat and/or previously documented associations with MBF variables in this population [16], [17]. Interaction terms were examined to determine whether the associations with MBF measures were modified by gender or race/ethnicity. There was a significant gender interaction for resting MBF (p = 0.001), but not hyperemic MBF (p = 0.37) or PR (p = 0.13). There were no interactions with race/ethnicity (p>0.10). Based on these findings, gender-stratified models were adjusted for age, race/ethnicity, education, smoking, LDL and HDL cholesterol, statins, hormone replacement therapy (HRT, in women only), C-reactive protein, diabetes, diastolic blood pressure, anti-hypertensive medications, and coronary calcium. The presence of coronary calcium was defined as an Agatston score >0; however, we also considered scores ≥100 and ≥400. To determine if the associations were independent of obesity or cardiac work, we additionally adjusted for BMI, waist circumference, waist-to-hip ratio, and RPP, in separate models. Logistic regression was used to estimate odds ratios (OR) and 95% confidence intervals (CI) for a reduced PR, defined as <2.5 [30], [31]. Associations with MBF measures are reported per 1-standard deviation (SD) increment in pericardial fat (57.5 cm^3^ for men and 31.3 cm^3^ for women). Statistical significance was set at p≤0.05.

## Results

### Participant Characteristics

Participant characteristics by gender are shown in Table 1. The prevalences of diabetes (p = 0.04) and coronary calcium (p = 0.0002) were higher in men compared to women, while more women had abdominal obesity (45% vs. 72% based on a waist circumference >88 cm in women and >102 cm in men, p<0.0001) and reported smoking <100 cigarettes in their lifetime (p = 0.04). Women had a lower waist circumference, waist-to-hip ratio, left ventricular mass, and diastolic blood pressure than men, but higher HDL cholesterol and C-reactive protein (p<0.0001 for all). Women also had less pericardial fat than men (p<0.0001), but not after normalizing to left ventricular mass (p = 0.28). MBF and CVR at rest and during hyperemia (p<0.0001 for all), as well as PR (p = 0.03) were higher in women.

**Table 1:** Participant characteristics.

Characteristic	Men (n = 121)	Women (n = 93)
Age (yrs)	60.4±10.4	59.5±10.3
Hispanic (%)	47.9	37.8
Education		
High school diploma or GED	34.7	41.9
Some college or technical school	35.5	30.1
Bachelor's or graduate degree	29.8	28.0
Smoking status (%)		
Never	33.1	48.4*
Former	51.2	34.4
Current	15.7	17.2
Diabetes (%)	14.1	5.4*
Hypertension (%)	31.4	28.0
Coronary calcium score (%)		
0	34.7	62.4*
0.01–99	28.9	23.7
100–399	19.0	8.6
≥400	17.4	5.4
Systolic blood pressure (mmHg)	122±15	119±22
Diastolic blood pressure (mmHg)	75±8	65±10†
Heart rate (beats/min)	62±10	63±9
HRT use (%)	—	43.0
Statin use (%)	14.9	11.8
HDL cholesterol (mg/dl)	43.6±11.9	53.9±12.8†
LDL cholesterol (mg/dl)	120.5±28.2	115.2±28.3
C-reactive protein (mg/l)	1.42 (0.70–2.96)	2.99 (1.43–7.24)†
BMI (kg/m^2^)	28.8±4.2	28.9±5.4
Waist circumference (cm)	100.8±11.5	97.2±14.1†
Waist-to-hip ratio	0.097±0.064	0.090±0.073†
Pericardial fat (cm^3^)	105.2±57.5	71.9±31.3†
Pericardial fat index (cm^3^/g)	0.59±0.32	0.55±0.23
Left ventricular mass (g)	183.2±35.3	132.0±29.0†
Resting MBF (ml/min/g)	0.93±0.19	1.12±0.23†
Hyperemic MBF (ml/min/g)	2.65±0.72	3.49±0.76†
Perfusion reserve	2.93±0.89	3.19±0.78*
Resting RPP (beats/min mmHg)	0.90±0.17	0.92±0.25
Hyperemic RPP (beats/min mmHg)	1.06±0.23	1.12±0.30
Resting CVR (mmHg/ml/min/g)	110.98±23.00	85.73±16.85†
Hyperemic CVR (mmHg/ml/min/g)	37.85±12.33	27.11±9.85†

Table values are mean ± SD or median (interquartile range). Significant gender difference,

*p<0.05,

† p<0.0001.

BMI = body mass index; CVR = coronary vascular resistance; HRT = hormone replacement therapy; HDL = high-density lipoprotein; LDL = low-density lipoprotein; MBF = myocardial blood flow; RPP = rate-pressure product.

### Associations with Pericardial Fat

Pericardial fat was higher in obese (BMI≥30 kg/m^2^) vs. non-obese men (141.5±62.8 cm^3^ vs. 83.7±41.4 cm^3^, p<0.0001) and women (91.6±31.3 cm^3^ vs. 58.9±23.7 cm^3^, p<0.0001). Pericardial fat was also higher in men and women with abdominal obesity (134.8±64.2 cm^3^ and 81.4±30.1 cm^3^, respectively) compared to those without (81.4±37.4 cm^3^ and 44.8±14.3 cm^3^, respectively, p<0.0001 for both). These differences remained after normalizing pericardial fat to left ventricular mass (p<0.01 for all). In both men and women, pericardial fat was positively associated with age, C-reactive protein, BMI, waist circumference, waist-to-hip ratio, heart rate, and resting RPP, and inversely associated with HDL cholesterol (Table 2). In women only, pericardial fat was also positively associated with blood pressure, left ventricular mass, resting MBF, and hyperemic RPP and inversely associated with PR. There were no significant associations between pericardial fat and LDL cholesterol, coronary calcium, CVR, or hyperemic MBF in either gender.

**Table 2:** Association between clinical and hemodynamic characteristics and pericardial fat.

Characteristic	Men (n = 121)	Women (n = 93)
Age (yrs)	0.23*	0.26*
Systolic blood pressure (mmHg)	0.13	0.48†
Diastolic blood pressure (mmHg)	0.12	0.24*
HDL cholesterol (mg/dl)	−0.42†	−0.41†
LDL cholesterol (mg/dl)	0.04	−0.05
Heart rate (beats/min)	0.44†	0.31*
C-reactive protein (mg/l)	0.37†	0.33*
Coronary calcium score	0.17	0.02
BMI (kg/m^2^)	0.60†	0.64†
Waist circumference (cm)	0.57†	0.65†
Waist-to-hip ratio	0.61†	0.47†
Left ventricular mass (g)	0.14	0.33*
Resting MBF (ml/min/g)	0.05	0.29*
Hyperemic MBF (ml/min/g)	−0.06	0.13
Perfusion Reserve	−0.10	−0.21*
Resting RPP (beats/min mmHg)	0.21*	0.37*
Hyperemic RPP (beats/min mmHg)	0.05	0.25*
Resting CVR (mmHg/ml/min/g)	−0.01	−0.16
Hyperemic CVR (mmHg/ml/min/g)	0.07	0.02

Table values are Spearman correlation coefficients (r).

*p<0.05,

† p≤0.0001;

Abbreviations as in Table 1.

### Pericardial Fat and MBF

In models adjusted for age, race/ethnicity, education, smoking, LDL and HDL cholesterol, statins, HRT, C-reactive protein, diabetes, diastolic blood pressure, anti-hypertensive medications, and coronary calcium, pericardial fat (per 1-SD increment) was positively associated with resting MBF in women (β = 0.083±0.031, p = 0.008, Table 3), but not in men (β = 0.016±0.021, p = 0.46; p = 0.01 for pericardial fat×gender interaction). Pericardial fat remained associated with resting MBF in women after further adjusting for waist circumference (p = 0.03) or waist-to-hip ratio (p = 0.01); however, additional adjustment for BMI (p = 0.12) or resting RPP (p = 0.07) attenuated this association. Pericardial fat was not associated with hyperemic MBF in neither men (β = 0.042±0.073, p = 0.56) nor women (β = 0.067±0.105, p = 0.52). Results were similar with and without adjustment for resting MBF. There was also no significant association between pericardial fat and PR in men (β = −0.002±0.088, p = 0.98), while the association in women approached significance (β = −0.147±0.094, p = 0.12). Using more stringent criteria to define the presence of coronary calcium (<100 vs. ≥100 or <400 vs. ≥400) had no major effect on the results (data not shown).

**Table 3:** Association between MBF measures and pericardial fat after multivariate adjustment.

Model	Men (n = 121)		Women (n = 93)	
	β±SE	P-value	β±SE	P-value
Resting MBF (ml/min/g)^a^	0.016±0.021	0.46	0.083±0.031	0.008
Plus BMI	0.030±0.025	0.23	0.059±0.037	0.12
Plus waist circumference	0.024±0.024	0.31	0.074±0.034	0.03
Plus waist-to-hip ratio	0.024±0.024	0.33	0.081±0.031	0.01
Plus resting RPP	−0.013±0.018	0.47	0.044±0.024	0.07
Hyperemic MBF (ml/min/g)^a^	0.042±0.073	0.56	0.067±0.105	0.52
Plus BMI	0.062±0.070	0.38	0.070±0.127	0.58
Plus waist circumference	0.069±0.084	0.42	0.090±0.119	0.45
Plus waist-to-hip ratio	0.005±0.081	0.95	0.062±0.108	0.56
Plus hyperemic RPP	0.029±0.070	0.68	−0.013±0.101	0.89
Perfusion Reserve^a^	−0.002±0.088	0.98	−0.147±0.094	0.12
Plus BMI	−0.019±0.102	0.85	−0.040±0.112	0.72
Plus waist circumference	−0.033±0.098	0.74	−0.086±0.106	0.45
Plus waist-to-hip ratio	−0.056±0.099	0.57	−0.146±0.097	0.14
Plus hyperemic RPP	−0.0004±0.089	1.00	−0.162±0.098	0.10

Table values are regression coefficients (β) ± standard errors (SE) per 1-SD unit increment in pericardial fat: 57.5 cm^3^ in men and 31.3 cm^3^ in women.

a Model is adjusted for age, race/ethnicity, education, smoking, LDL and HDL cholesterol, statins, diastolic blood pressure, anti-hypertensive medications, HRT (in women), C-reactive protein, diabetes, and coronary calcium. Abbreviations as in Table 1.

### Pericardial Fat and Reduced PR

**Figure 1:**
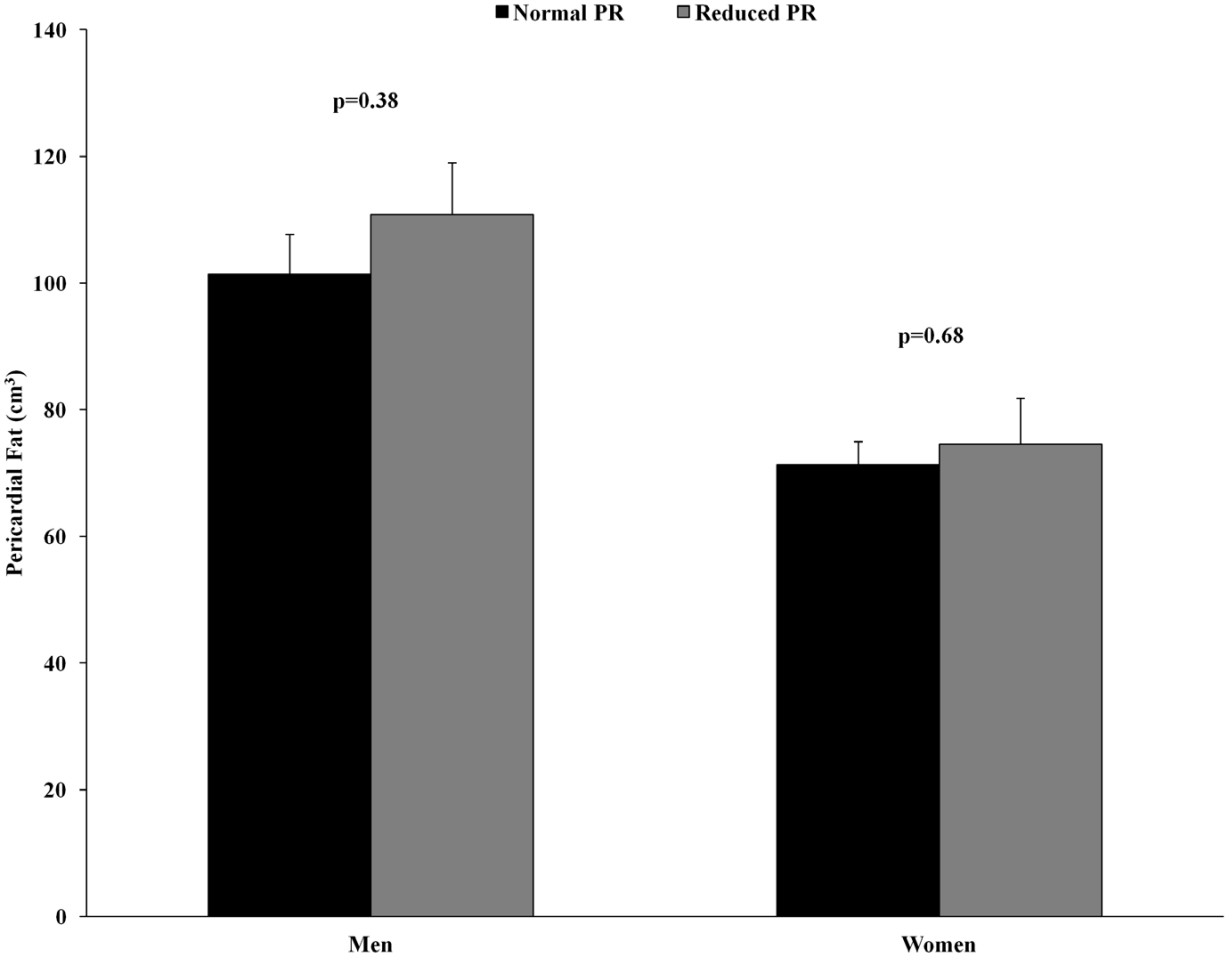
Pericardial fat in men and women with normal PR ≥2.5 and reduced PR <2.5.

The prevalence of a reduced PR (<2.5) was 2-fold greater in men compared to women (40.5% vs. 20.4%, p = 0.002). Pericardial fat averaged 110.8±8.2 cm^3^ and 101.4±6.3 cm^3^ in men (p = 0.38) and 74.6±7.2 cm^3^ and 71.3±3.7 cm^3^ in women (p = 0.68) with and without a reduced PR, respectively, as shown in Figure 1. Logistic regression analyses revealed no association between a 1-SD unit increment in pericardial fat and a reduced PR in men (OR, 1.18; 95% CI, 0.82–1.70) or women (OR, 1.11; 95% CI, 0.68–1.82).

## Discussion

We investigated whether pericardial fat is adversely related to myocardial perfusion in asymptomatic adults with no prior history of cardiovascular disease. The main finding of this study was that pericardial fat is not associated with coronary vasoreactivity. The lack of a significant relationship between pericardial fat and both hyperemic MBF and PR was surprising given that inverse associations have been found with cardiac obesity in symptomatic individuals [14], [15]. We did find an association between higher pericardial fat and higher resting MBF in women; however, this association was attenuated after further adjustment for BMI or RPP. Thus, our data fail to support an independent association between pericardial fat and myocardial perfusion in this population.

Previous studies investigating the association between cardiac obesity and myocardial perfusion have predominately included symptomatic individuals. Sade et al. reported that among women with angiographically normal coronary arteries, those with an impaired PR had 38% greater epicardial fat thickness on the free wall of the right ventricle than women with a normal PR [15]. However, measurements of epicardial fat thickness at a single point are highly dependent on cardiac anatomy and fat distribution [32] and do not correlate well with either epicardial or pericardial fat volume [20]. More recently, Bucci et al. found that among patients with obstructive CAD, epicardial fat volume was roughly 20% higher in those with a hyperemic MBF value below vs. above the median (≤1.75 ml/min/g) [14]. Additionally, higher epicardial fat was an independent predictor of lower hyperemic MBF and PR in multiple regression analyses. Janik et al. also reported that among patients presenting with angina and no prior cardiovascular disease, individuals with mild-to-severe ischemia had 38% higher epicardial fat volume compared to those with no ischemia [33]. In a similar study that included adults with and without symptoms, Tamarappoo et al. reported 22% higher pericardial fat volume and 24% higher epicardial fat volume in patients with ischemia compared to non-ischemic controls [13]. Although the proportion of asymptomatic individuals was fairly similar between cases and controls (63% vs. 54%, respectively), the presence of symptoms was one of the strongest predictors of prevalent ischemia in multivariable analyses, second only to epicardial/pericardial fat volume.

In contrast to the study by Tamarappoo et al., we were unable to find a significant association between pericardial fat and myocardial ischemia. In fact, pericardial fat was only 9% and 5% higher in men and women, respectively, with impaired PR compared to those with a normal PR. In addition, although we observed a significant correlation between higher pericardial fat and lower PR (in women only), this association was attenuated after adjusting for other risk factors. As such, these data suggest that pericardial fat does not have independent effects on myocardial perfusion in asymptomatic adults. Moreover, our findings highlight potentially important distinctions between asymptomatic and symptomatic persons with respect to subclinical atherosclerosis. For example, in the study by Tamarappoo et al., approximately 91% of the population had moderate-to-severe atherosclerosis as evidenced by coronary calcium scores ≥100 [13]. On the other hand, only 26% of our study participants had coronary calcium scores ≥100, with nearly half having no coronary calcium present at all. High coronary calcium scores are associated with a higher likelihood of significant coronary stenosis, whereas the absence of coronary calcium is associated with a very low likelihood of obstructive CAD [34], [35]. Moreover, symptomatic CAD patients with elevated coronary calcium scores have more severe stenosis than asymptomatic CAD patients with similar calcium scores [36], suggesting that the presence of symptoms does indeed reflect the underlying pathology, and likely the patient profile. In this regard, the prevalence of impaired coronary vasoreactivity (as defined using a lower cut-off value of PR<2.0) was very low in our women (6%), compared to those in the study by Sade et al. (40%). Furthermore, while the majority of our participants had a low-to-medium 10-year CAD risk (Framingham risk score = 8%), Tamarappoo et al. and Janik et al. investigated persons with Framingham risk scores of ∼12–14% [13], [33].

Obesity-related cardiovascular disease may be partially caused by altered adipokine-mediated signaling between local fat depots and the adjacent blood vessels and cardiomyocytes [37]. Epicardial fat has a high expression of chemokines and inflammatory cytokines [8], and increased periaortic fat in aging and obesity promotes vascular smooth muscle cell growth [9]. Thus, in the presence of excess fat, these pro-inflammatory activities are likely to be increased and thereby promote the development of vascular dysfunction and atherosclerosis. Consistent with this, Bucci et al. reported that among patients with CAD, only those with significant flow-limiting stenosis had increased epicardial fat volume, which suggests that in our population the combination of CAD and increased pericardial fat may promote impaired coronary vascular function, while CAD plus low/normal pericardial fat may not. Although we do not have direct measures of CAD, we did find that the prevalence of a reduced PR was 48% in participants with coronary calcium present and pericardial fat volume in the highest quartile (≥130.5 cm^3^ in men, ≥91.2 cm^3^ in women), while the prevalence was only 15% in participants with no coronary calcium and pericardial fat in the lowest quartile (<64.0 cm^3^ in men, <47.6 cm^3^ in women). We also found positive associations between pericardial fat and C-reactive protein, age, blood pressure, HDL cholesterol, BMI, waist circumference, and left ventricular mass in this study, which confirms previous findings in the Framingham Heart Study [38]. Although a significant univariate association was found between pericardial fat and resting MBF in women, adjusting for BMI attenuated this relationship. Resting RPP (an indicator of cardiac work) also appeared to be an important determinant of resting MBF and accounted for much of the association between pericardial fat and resting MBF. It is important to remember that our measurement of pericardial fat reflects both the direct paracrine effects of epicardial fat on the coronary arteries, as well as the indirect systemic effects of thoracic visceral fat (i.e. paracardial fat) on metabolic risk factors [39]. Thus, taken together, these data suggest that in asymptomatic individuals, global obesity may have a greater influence on coronary vasoreactivity than cardiac obesity.

There are a few limitations in this study. The sample size was relatively small, which may have limited our ability to detect associations in men vs. women. Although gender differences in the prevalence and severity of cardiovascular diseases are well-documented, the impact of gender on obesity-related changes in MBF requires further study. In addition, our investigation was limited to cross-sectional analyses, which cannot determine whether increased pericardial fat precedes coronary vascular dysfunction. Similarly, our assessment of abdominal obesity was limited to anthropometric measures that cannot distinguish between visceral and subcutaneous fat. Without direct measures of total and abdominal fat, however, the relative importance of pericardial fat remains to be elucidated. We also cannot rule out the possibility that our participant subset is not completely representative of or generalizable to the larger MESA population, nor can we confirm the absence of obstructive atherosclerotic lesions since our participants did not undergo coronary angiography. Finally, although the myocardial perfusion measurements are fairly reproducible, the variability of the hyperemic MBF response over 1 year (absolute repeatability coefficient = 1.19 ml/min/g) has been shown to increase with the length of time between baseline and follow-up measurements [22]. This bias may underestimate the true variability in hyperemic MBF over longer periods of time, which is important to know for prospective population-based studies designed to assess the influence of risk factors on disease incidence and progression.

In conclusion, pericardial fat is not independently associated with hyperemic MBF or PR in asymptomatic men and women with no prior history of cardiovascular disease. These results are in contrast to a previous study in predominately symptomatic adults. Despite the present negative findings, our study provides some insight into the relationship among pericardial fat, atherosclerosis, and MBF in asymptomatic vs. symptomatic individuals. In this regard, it seems plausible that individuals with and without ischemic symptoms may have a different subclinical atherosclerotic disease burden, which may influence the effect of pericardial fat on coronary microvascular function. These differences may have important clinical implications for improving risk stratification in asymptomatic populations. Given the growing evidence that pericardial fat may be an important therapeutic target in the prevention of CAD [4], [5], further research in this area is warranted.
